# Successful reduction of recalcitrant ileocolic intussusception with a double-balloon-tipped catheter that prevents air leaks and catheter expulsion

**DOI:** 10.1016/j.radcr.2021.07.063

**Published:** 2021-08-28

**Authors:** Richard H. Jones

**Affiliations:** Department of Radiology and Radiological Science, Division of Pediatrics, Department of Pediatrics, Division of General Pediatrics, Medical University of South Carolina, 96 Jonathan Lucas St, CSB HE210C MSC 323, Charleston SC 29425, USA

**Keywords:** Ileocolic intussusception, Reduction, Delayed repeat enema, Enema, Double balloon catheter

## Abstract

Noninvasive reduction of ileocolic intussusception requires increasing intracolonic pressure via gas or liquid administered through a rectal catheter. A tight seal around the catheter is required to maintain intracolonic pressures and this tight seal is difficult to maintain with existing techniques. I describe the safe and effective use of a catheter with 2 balloons near the tip that surround the anus internally and externally to prevent leakage of air during an enema on a toddler after failure with a single-balloon tipped catheter.

## Introduction

Ileocolic intussusception is the telescoping of the ileum into the colon and is most commonly diagnosed in pediatric patients [1]. Diagnosis of ileocolic intussusception via abdominal ultrasound is accurate [2] and its treatment by colonic pressurization under image guidance is one of the few emergency procedures performed non–interventional radiologists [3]. The exasperating problem of a poor seal or leakage of air or fluid around the rectal catheter and out the anus has received little to no attention in the literature and this prolongs procedural time and radiation dose in the author's experience. Some children expel the catheter with or without the balloon inflated during reduction attempts, which also prolongs procedure time and radiation dose. A double-balloon tipped catheter (SealCath LLC, Mount Pleasant, SC www.sealcath.com) was developed to avoid these difficulties. Herein I describe the use of this catheter during the reduction of an intussusception that initially failed because of air leakage and expulsion of the catheter.

## Case report

An otherwise healthy 2-year-old African-American female presented to the emergency department with episodic abdominal pain for 12 hours. There were no bloody stools, diarrhea, or vomiting. There were no symptoms of recent upper respiratory infection. Physical exam showed a nontender abdomen without palpable mass.

**Fig. 1:**
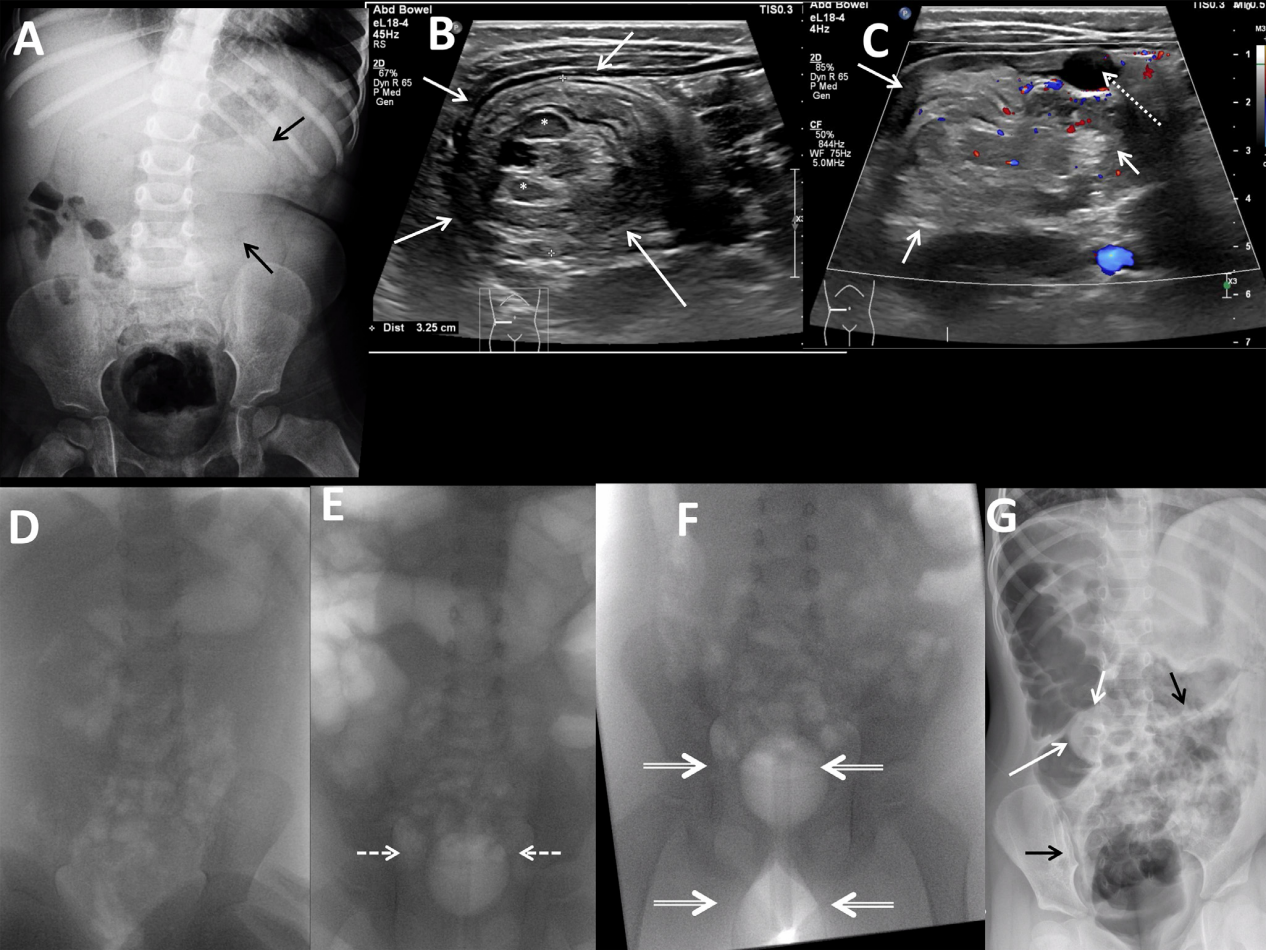
Two-year-old female with ileocolic intussusception and unsuccessful reduction. (A) Frontal supine radiograph of the abdomen shows a suggestion of a tubular soft tissue mass in the midabomen (black arrows). (B, C) Linear transducer grayscale transverse image of the right midabomen shows a 3.2 cm diameter round mass (white arrows) containing lymph nodes (white asterisks), echogenic fat, bowel, and alternating concentric layers of echogenic tissue and internal color Doppler signal. Gallbladder noted (dotted white arrow). (D) Frontal supine fluoroscopic image of the abdomen prior to air insufflation shows minimal bowel gas. (E) Frontal supine fluoroscopic image of the abdomen with 30 mL air in 20 Fr Foley catheter balloon (white dashed arrows). All intrarectal air is displaced by the balloon. (F) Frontal prone fluoroscopic image of the abdomen with 50 mL air in internal balloon, 70 mL air in external balloon (white double arrows). All intrarectal air is displaced by the balloon. (G) Left side down lateral decubitus view after attempted intussusception reduction shows rounded filling defect at the ileocecal valve (white arrows), increased air in the cecum and central abdominal bowel loops (black arrows), and no pneumoperitoneum.

An abdominal ultrasound identified ileocolic intussusception in the right and transverse colon (Figs. 1B and C). Color Doppler signal was present within the intussusception, and there was no entrapped fluid or lead point mass. A supine radiograph identified a tubular mass in the expected location of the transverse colon representing the intussusception (Fig. 1A). There was no preprocedural pneumoperitoneum.

Prior to the reduction attempt, an intravenous catheter was placed, surgical consult was obtained, a nurse accompanied the patient to the fluoroscopy suite to monitor the patient during reduction, and written consent for reduction was obtained from the patient's mother, who also accompanied the patient. The patient was not sedated.

A lubricated 20 Fr Foley catheter (Fig. 1E) was inserted into the anus and the retention balloon was inflated with 30 mL of air in the rectum under fluoroscopic visualization. Hand-sphygmomanometer insufflation of air through the catheter through a 3-way connector with a pressure release valve in supine and prone positions was capable of reducing the intussusception to the ileocecal valve under fluoroscopic guidance but air could not be refluxed into the terminal ileum. Air leaks prevented further reduction as intracolonic pressures approached 80 mm Hg, and the patient expelled the catheter with the balloon inflated despite manual pressure held on the tube and buttocks. Residual intussusception at the ileocecal valve was confirmed with ultrasound. An external tape plug and anal occlusion disc were then placed around the exterior side of the Foley catheter, the internal balloon was inflated with 30 mL of air, and the catheter was taped to the buttocks. Again, air leaks at 80 mm Hg disallowed further reduction, and the patient expelled the catheter.

Next, a lubricated 30 Fr double balloon-tipped catheter (Fig. 3) was placed per rectum (Fig. 1F). The internal balloon inflated with 50 mL air, and the external balloon inflated with 70 mL air (the maximum volumes recommended by the manufacturer). During insufflation the patient expelled the catheter 3 times and the procedure was concluded. A filling defect remained at the ileocecal valve (Fig. 1G) and there was no post-procedural pneumoperitoneum. Total procedural time was 1 hour. The patient was admitted for observation. Surgery consultation recommended repeating ultrasound if symptoms of episodic abdominal pain recurred. The patient resumed a normal diet and activities.

**Fig. 2:**
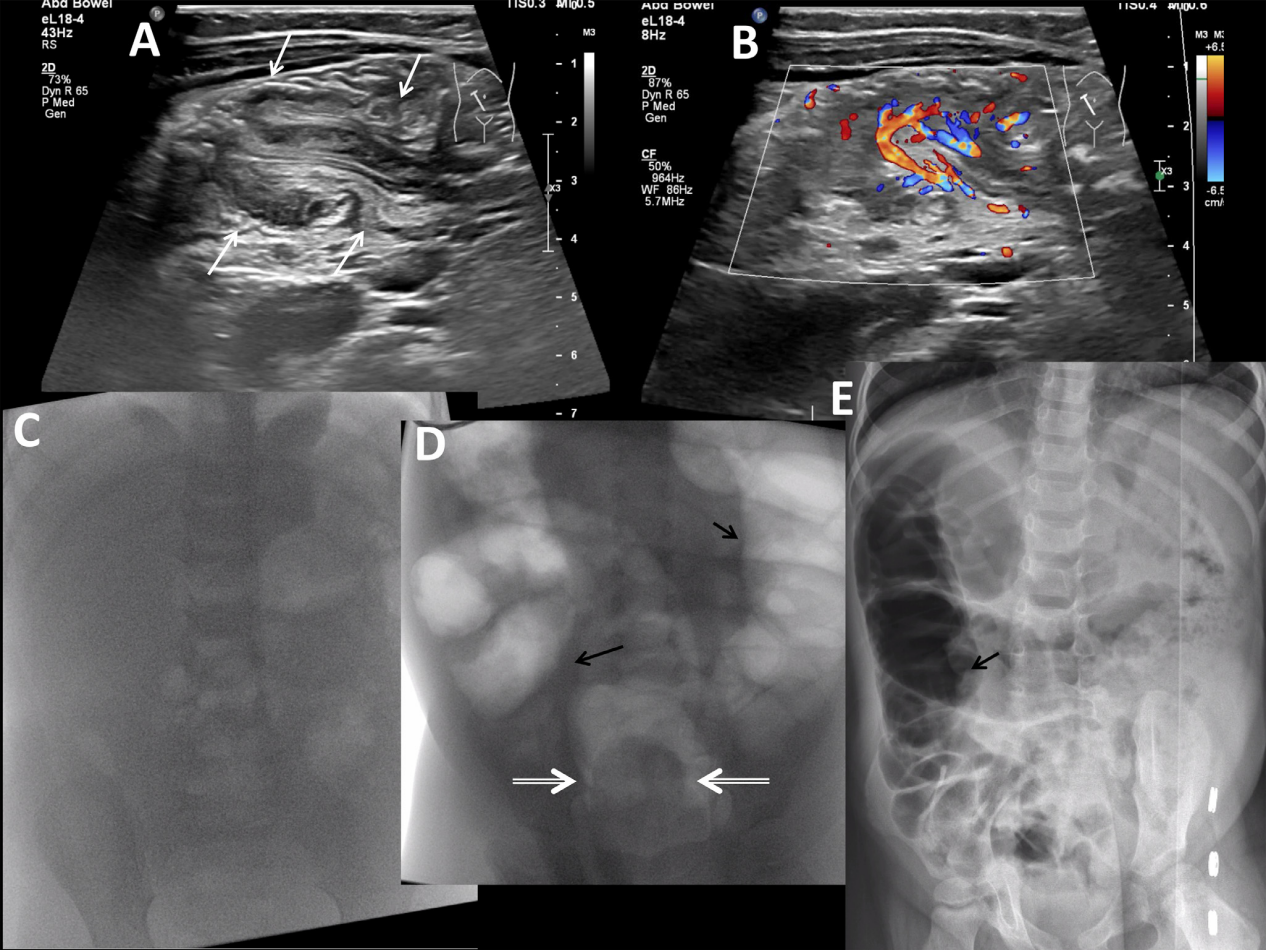
Two-year-old female with ileocolic intussusception and successful reduction. (A, B) Linear transducer grayscale (A) and color (B) Doppler images of the right midabdomen show intussusception in long axis at the hepatic flexure as a linear structure with concentric alternating bands of echogenicity representing bowel within bowel with presence of color Doppler signal. (C) Pre-reduction frontal prone fluoroscopic view of the abdomen shows minimal bowel gas. (D) Intraprocedural frontal prone fluoroscopic view of the abdomen shows the internal balloon filled with 50 mL water (double arrows). Some intrarectal air surrounds the lateral edges of the balloon, but not the caudal end. The air-filled external balloon is not included in the field of view. There is increased colonic air (black arrows) during air insufflation through the catheter. (E) Post-reduction left side down lateral decubitus view of the abdomen shows no filling defect within the air-filled cecum, increased central bowel gas (black arrows), and no pneumoperitoneum.

Eighteen hours later after the patient experience repeated symptoms of episodic abdominal pain, ultrasound identified recurrence of the ileocolic intussusception in the ascending colon (Figs. 2A and B) without lead point mass, entrapped fluid, or absence of Doppler signal.

**Fig. 3:**
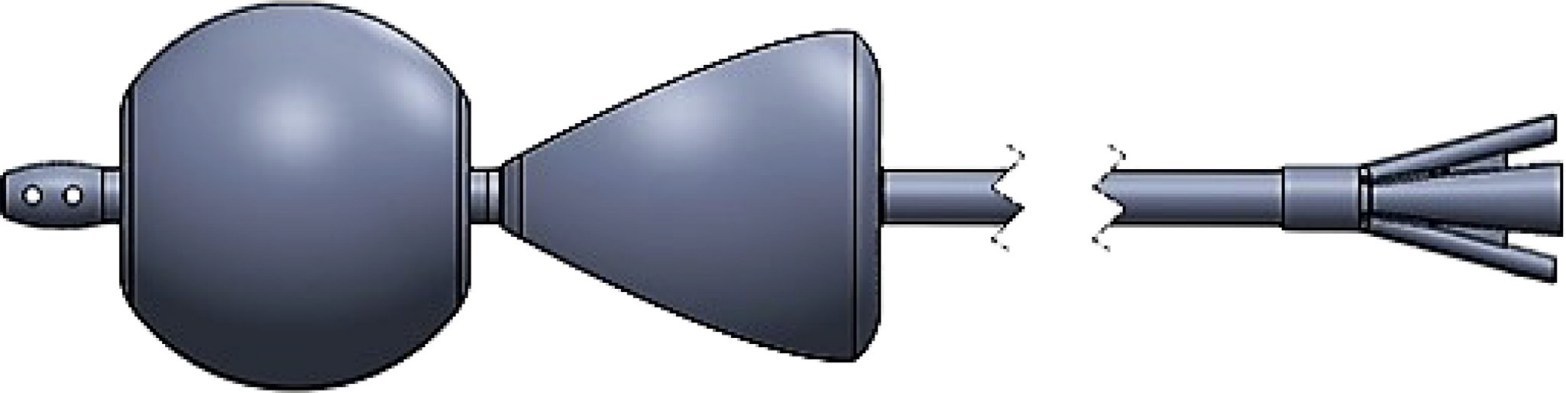
SealCath double balloon catheter schematic drawing. 30 Fr triple lumen catheter with internal and external balloons. www.sealcath.com.

The patient received 5 mg intravenous lorazepam per mother's preference for sedation, and was returned to the fluoroscopy suite after obtaining consent for intussusception reduction, again accompanied by her mother and a nurse for intraprocedural monitoring. A lubricated 30 Fr double balloon-tipped catheter was placed per rectum. The internal was balloon inflated in the rectum under fluoroscopy with 50 mL water and external balloon inflated with 70 mL air. Hand-sphygmomanometer insufflation with the patient in the prone position achieved sustained intracolonic pressures of 80-100 mm Hg with a notable reduction in rectal air leakage. Air was refluxed into the terminal ileum (Fig. 2E) and there was no filling defect at the ileocecal valve. There was no post-procedural pneumoperitoneum. Total procedural time from insertion to removal of the catheter was 5 minutes. The patient was discharged home the same day and has had no further procedures or symptoms as of this writing.

## Discussion

Ileocolic intussusception is the telescoping of the ileum into the large bowel and places the affected bowel at risk for ischemia and necrosis. Surgical reduction and/or resection of the intussuscepted segment of bowel and colon is necessary if there is a bowel perforation, if there is a lead point mass such as a bowel neoplasm or Meckel diverticulum, or if non–invasive reduction fails. However, noninvasive reduction is the standard of care. Delayed repeat enemas (as in this case) may be used for failed initial reduction with rates of bowel perforation 1% or less [1]. Unsuccessful noninvasive reduction and need for surgical treatment is more likely when a bowel obstruction is present on preprocedural radiographs [4]. Close collaboration between surgeons and radiologists at pediatric referral centers is required for safe and effective treatment.

Various methods of non–invasive ileocolic intussusception reduction have been described including hand-sphygmomanometer insufflation of air [5], fluid contrast via gravity under fluoroscopic guidance, or with fluid contrast under ultrasound guidance [6]. There is a strong preference for air reduction in a recent survey of pediatric radiologists predominantly in North America [7]. All these methods require maintenance of sustained increased intracolonic pressure and the major difficulty in sustaining this pressure is preventing air leak from the rectum. Air leaks are a common complication of ileocolic intussusception reduction and manifest as (1) dropping colonic pressure on the sphygmomanometer during insufflation, (2) audible passage of air per rectum, (3) intermittent reduction in size of air-filed colon on fluoroscopy. Anecdotally, the practitioner's forearm muscles used for squeezing the insufflation bulb tire quickly due to leaks and a second person is often needed to squeeze the bulb.

Preventing air leaks can be challenging, even with a balloon-tipped catheter that occupies the whole volume of the rectum, wrapping a tape plug around the end of the tube, taping of the tube against the buttocks, using an external anal disc occlusion device, manual pressure squeezing the buttocks together, and prone positioning [8]. Of these techniques, taping is used most commonly [7]. Internal balloons have been shown to be effective [9] but have a trend toward increased perforation when used at 9 months’ of age or younger [10]. Other than a catheter with a balloon, these other tube modifications to prevent leaks require additional time and their use and proficiency vary between practitioners. Additional personnel, usually 1 or 2 people, are specifically needed just to prevent leaks (ie one person who holds the buttocks together and another to hold the catheter in place).

Even if air leaks can be prevented with these maneuvers, insufflation of the colon cannot reduce an intussusception if the patient expels the catheter and/or internal balloon. This complication has received almost no attention in the literature. This problem may be more common in older or less critically-ill or non–sedated patients who are able to bear down with considerable force. Sedation is uncommonly used during this procedure [7] and its effect on reduction is controversial [11] and on balloon expulsion not studied. It has been proposed that atropine reduces the time necessary to complete an ileocolic intussusception due to its smooth-muscle relaxing properties [12] however the effect on balloon expulsion is also not studied.

The double-balloon catheter was developed to prevent air leaks by a third party (SealCath LLC, Mount Pleasant, SC,www.sealcath.com). This catheter most effectively prevented air leaks with maximal inflation of the internal and external balloons (50 mL and 70 mL, respectively). The internal balloon is most effective when it fully occupies the rectum, and must be inflated before the external balloon. As the external balloon inflates it more forcefully pushes the internal balloon against the anus between the 2 balloons, improving the seal. The minimal balloon volumes necessary to achieve the best seal while not over pressurizing the rectum vary between patients. Appropriate sizing can be accomplished by visual evaluation of the balloons under fluoroscopy. Once the catheter is inserted, the internal balloon size can be easily determined using air or liquid contrast and manual feedback, but the internal balloon will be difficult to see if filled with water when the rectum does not contain air. A lateral decubitus view is my preferred projection when using this catheter to provide the best assessment of relative rectal and balloon size, with volume adjustments made during insufflation as necessary if leaks occur. In my experience leaks are very infrequent and adjustments are often not necessary.

The double balloon catheter could be expelled from the rectum while making a tight seal. The first attempt failed because air was used for the internal balloon, and the balloon was thus more compressible and therefore more easily squeezed through the anus and expelled than when it was filled with water on the second, successful attempt. I recommend fluid (water or contrast) in the internal balloon and air or water in the external balloon if expulsion occurs.

In summary this new catheter was successful when used for a delayed repeat enema in treating ileocolic intussusception in a 2-year-old female because its double balloon design prevented leakage of air and disallowed balloon expulsion. Because of its ease of use (ie no taping or other tube modifications) and generally shorter procedural times we have moved toward using this catheter during initial treatment of ileocolic intussusception at our institution. The catheter has other potential uses in diagnostic contrast enemas or fistulagrams, or other instances where preventing a leak at an orifice or stoma is helpful.

Further studies to assess the safety, effectiveness, and ideal balloon sizes and contents of double-balloon tipped catheters for the reduction of ileocolic intussusception are indicated. A better seal and higher sustained pressures may increase the likelihood for perforation, although perforation is rare with air reduction using existing catheters when used in combination with a pressure-release valve.

## Patient consent

Consent was obtained from the patient's parent for publication of this case report.
